# A Comparison Between Recombinant *Listeria* GAPDH Proteins and GAPDH Encoding mRNA Conjugated to Lipids as Cross-Reactive Vaccines for *Listeria, Mycobacterium*, and *Streptococcus*

**DOI:** 10.3389/fimmu.2021.632304

**Published:** 2021-04-19

**Authors:** Hector Teran-Navarro, David Salcines-Cuevas, Ricardo Calderon-Gonzalez, Raquel Tobes, Jorge Calvo-Montes, Inmaculada Concepción Pérez-Del Molino Bernal, Sonsoles Yañez-Diaz, Manuel Fresno, Carmen Alvarez-Dominguez

**Affiliations:** ^1^Instituto de Investigación Marqués de Valdecilla (IDIVAL), Santander, Spain; ^2^Grupo de Oncología y Nanovacunas, Santander, Spain; ^3^Alamo Blanco, Granada, Spain; ^4^Microbiology Department, Hospital Universitario Marqués de Valdecilla, Santander, Spain; ^5^Dermatology Department, Hospital Universitario Marqués de Valdecilla, Santander, Spain; ^6^Facultad de Medicina, Universidad de Cantabria, Santander, Spain; ^7^DIOMUNE S.L., Parque Científico de Madrid, Madrid, Spain; ^8^Centro de Biología Molecular Severo Ochoa, Universidad Autónoma de Madrid, Madrid, Spain; ^9^Facultad de Educación y Facultad de Ciencias de la Salud, Universidad Internacional de La Rioja, Logroño, Spain

**Keywords:** glyceraldehyde-3-phosphate-dehydrogenase, listeriosis, pneumonia, tuberculosis, cross-reactive vaccines, innate immunity

## Abstract

Cross-reactive vaccines recognize common molecular patterns in pathogens and are able to confer broad spectrum protection against different infections. Antigens common to pathogenic bacteria that induce broad immune responses, such as the glyceraldehyde-3-phosphate dehydrogenase (GAPDH) of the genera *Listeria, Mycobacterium*, or *Streptococcus*, whose sequences present more than 95% homology at the N-terminal GAPDH_1−22_ peptide, are putative candidates for universal vaccines. Here, we explore vaccine formulations based on dendritic cells (DC) loaded with two molecular forms of *Listeria monocytogenes* GAPDH (LM-GAPDH), such as mRNA carriers or recombinant proteins, and compare them with the same molecular forms of three other antigens used in experimental vaccines, listeriolysin O of *Listeria monocytogeness*, Ag85A of *Mycobacterium marinum*, and pneumolysin of *Streptococcus pneumoniae*. DC loaded with LM-GAPDH recombinant proteins proved to be the safest and most immunogenic vaccine vectors, followed by mRNA encoding LM-GAPDH conjugated to lipid carriers. In addition, macrophages lacked sufficient safety as vaccines for all LM-GAPDH molecular forms. The ability of DC loaded with LM-GAPDH recombinant proteins to induce non-specific DC activation explains their adjuvant potency and their capacity to trigger strong CD4^+^ and CD8^+^ T cell responses explains their high immunogenicity. Moreover, their capacity to confer protection in vaccinated mice against challenges with *L. monocytogenes, M. marinum*, or *S. pneumoniae* validated their efficiency as cross-reactive vaccines. Cross-protection appears to involve the induction of high percentages of GAPDH_1−22_ specific CD4^+^ and CD8^+^ T cells stained for intracellular IFN-γ, and significant levels of peptide-specific antibodies in vaccinated mice. We concluded that DC vaccines loaded with *L. monocytogenes* GAPDH recombinant proteins are cross-reactive vaccines that seem to be valuable tools in adult vaccination against *Listeria, Mycobacterium*, and *Streptococcus* taxonomic groups.

## Introduction

Vaccines for adults is one of the biggest challenges of current vaccinology and several methodologies have been proposed for this purpose such as reverse vaccinology, a genome-based approach to vaccine development (1), or immune algorithm approaches (2–4). One of the main issues regarding vaccines for adults is the possibility to prepare bacterial vaccines that induce cross-protection against infections caused by different pathogens that provide cellular specific immunity, involving both T and B cells, known as cross-reactive vaccines (CRV). However, cross-protection against infections can also be achieved if innate immune cells acquire long functional states such as in trained immunity-based vaccines (TIbV) (5). Dendritic cells (DC) are pivotal cells for conventional, CRV, or TIbV vaccines and serve as efficient vaccine platforms. In this regard, DC based vaccines can recognize non-specific patterns in pathogens and can induce specific immunity (5–7), allowing cross-protection against infections. In fact, the COVID-19 pandemic has highlighted the possibility that vaccines designed for unrelated pathogens such as *Mycobacterium bovis Bacillus Calmette-Guérin* (BCG), could also confer some protection for a coronavirus (8, 9).

Bacterial pathogens such as *Mycobacterium tuberculosis, Listeria monocytogenes*, or *Streptococcus pneumonia* can cause severe meningitis both in the elderly and in adults with immunocompromising conditions, such as cancer patients, in all cases that require long-term antibiotic treatment (10). Opportunistic skin diseases, mild or severe, caused in adults by *Mycobacterium marinum, Mycobacterium chelonae, Mycobacterium fortuitum, Listeria monocytogenes*, or *Streptococcus pyogenes* also require long-term treatment with antibiotics that might contribute to the development of antibiotic resistance (11–13). On the other hand, there are no vaccines available for meningitis or severe skin diseases in the elderly (14). Preparing DC based vaccines that can cross-protect against bacterial genera of *Listeria, Mycobacterium*, or *Streptococcus* might therefore provide relevant tools for adult vaccination.

Poly-bacterial preparations such as MV130 (Bactek®) are composed of heat-inactivated bacteria with 90% gram-positive bacteria (*Streptococcus pneumoniae, Staphylococcus aureus, Staphylococcus epidermidis*) and 10% gram-negative bacteria (*Klebsiella pneumoniae, Haemophilus influenza*, and *Moraxella catarrhalis*) (15). MV130 acts as adjuvant and improves recurrent respiratory tract infections by inducing a specific T cell immunity against bacteria present in the preparation, but also with T cell responses to other different antigens (16, 17). The ability of MV130 to immunomodulate DC, implies the triggering of Toll-like (TLR) and Nod-like receptors (NLR) with the ability to stimulate Th1 and Th17 immune responses and increases the levels of IL-10 (18). Other bacterial adjuvants such as DIO-1, a lipopolysaccharide of *Ochrobactrum intermedium* that acts as a TLR-2/4 agonist, is also able to immunomodulate DC, inducing Th1 immune responses and conferring protection against experimental listeriosis in different vaccine formulations (19–21).

Bacterial ADP-ribosylating enterotoxins such as the heat-labile enterobacterial toxin subunit of *Escherichia coli* (LT), or the cholera toxin (CT) are also used as adjuvants as they promote multifaced antigen-specific responses inducing Th1, Th2, and Th17 patterns. The availability of LT and CT mutants lacking toxicity have allowed these bacterial toxins to be included in vaccine designs, as they retain their adjuvant capacities (22). Other bacterial enzymes with ADP-ribosylating abilities are the glyceraldehyde-3-phosphage dehydrogenases (GAPDH) of gram-positive bacteria, also proposed as universal vaccines against different *Streptococcus* serotypes, since they induce broad spectrum immune responses (23). Our group also described that the GAPDH of *L. monocytogenes* (GAPDH-LM, Lmo 2459), which also presents ADP-ribosylating abilities (24), showed two interesting abilities for vaccine designs—a 22 amino acid peptide at the N-terminal that presented 95–98% sequence homology to GAPDH of *Mycobacterium* and *Streptococcus* and the ability of anti-*Listeria* GAPDH antibodies to recognize *Mycobacterium* or *Streptococcus* spp (25–28).

Messenger RNA (mRNA) is a promising vehicle for vaccination (29), however, naked mRNA suffers a quick degradation by RNases activity and is consequently not internalized efficiently. Several delivery carriers for mRNA vaccines have been developed, mostly based on lipid particulate complexes. Typical examples are the COVID-19 vaccines by Moderna and Pfizer-BioNTech and others such as nanoparticles (30–33). In this regard, cationic lipids commercially available, such as lipofectamine (Invitrogen), can also serve as protective capsules to incorporate nucleic acids into eukaryotic cells. In fact, this is a classical procedure to transfect cDNA or antisense oligonucleotides into cells as well as showing antimicrobial abilities (34–36). In this study, we compare the immune response capacities of mRNA encoding GAPDH encapsulated in lipofectamine (mRNA-GAPDH-LIPO) and GAPDH recombinant proteins with antigens involved in experimental vaccines such as listeriolysin O (LLO) of *L. monocytogenes* (LM), Ag85A antigen of *M. marinum* (MM), or pneumolysin (PLY) of *S. pneumoniae* (SP) (37–42) and explore their potential as CRV vaccines to confer antigen cross-protection immunity.

## Materials and Methods

### Bacteria, Adjuvants, Cells, Reagents, and Cell Medium

We used *L. monocytogene*s wild type 10403S strain (LMWT) and LLO *L. monocytogenes* deficient mutant (LMΔLLO) derived from the 10403S strain (Prof. D.A. Portnoy, University of California, Berkley, CA, USA). The *Mycobacterium smegmatis* strain was donated by F.J. Sangari and A. Seoane (IBBTEC-University of Cantabria, Santander, Spain) and the *S. pneumoniae* non-pathogenic vaccine strain, 49619-19F, was obtained commercially from ATCC. *Listeria monocytogenes* (LM), *Mycobacterium marinum* (MM), *M. chelonae* (MC), *Mycobacterium avium* (MA), *Mycobacterium tuberculosis* (MTB), *Streptococcus pneumoniae* (SP) (all of them serotype 5), *Streptococcus pyogenes* (SPY), and *Streptococcus agalactiae* (SA) were all clinical isolates of the Microbiology Department at our institution (Hospital Universitario Marqués de Valdecilla, Santander, Spain). DIO-1 is a TLR2/4 targeted molecule that we used as an adjuvant (19–21). Bone-marrow-derived macrophages (DM) or bone-marrow-derived dendritic cells (DCs) were obtained from femurs of 8–12-week-old female mice. DMs or DCs were cultured at 2 × 10^6^ cells/mL in six-well-plates in Dulbecco's Modified Eagle's Medium (DMEM) supplemented with 20% fetal calf serum (FCS), 1 mM glutamine, 1 mM non-essential amino acids, 50 μg/mL gentamicin, and 30 μg/mL vancomycin (DMEM complete medium) and 20 ng/mL granulocyte–macrophage colony-stimulating factor (GM-CSF) for DC, was added to the complete medium to obtain differentiated immune cells. On Day 7, the cells were harvested and analyzed by fluorescence-activated cell sorting (FACS) to evaluate cell surface markers and appropriate differentiation of DCs using the following markers: CD11b–fluorescein isothiocyanate (FITC), CD11c–phycoerythrin (PE), IAb–allophycocyanin (APC), F4/80–PE, CD80–FITC, and CD86–V450 (BD Biosciences, Palo Alto, CA). Cells were collected using cell scrapers to detach adherent cells. In certain samples we also used, after detachment, anti-mouse CD11c-coated magnetic beads and MACSTM separation columns (Miltenyi Biotech Inc., Auburn, CA) on day 7 for positive selection, as previously described (34). Lipofectamine was obtained from Invitrogen.

### Mice

We used C57BL/6 mice from our animal facilities at the University of Cantabria at 20–24 weeks old, an age that mimics human beings that are 50 years old and older. LD50 of the *L. monocytogenes* strain 10403S in C57BL/6 mice is 2 × 105 CFU/mice (2, 39, 43). LD50 of LM (HUMV-01) was 2-fold higher 4 × 105 CFU/mice. LD50 of *M. marinum* (HUMV-MM01) is 2 × 104 CFU/mice in C57BL/6 mice and LD50 of *S. pneumoniae* (HUMV-SP01) is 5 × 104/mice in C57BL/6 mice. LD50 were evaluated in groups of mice (*n* = 10) i.v infected with 2 × 104 CFU/mice, 5 × 104 CFU/mice or 105 CFU/mice. Mice were examined for death every 12 h and checked for clinical parameters of illness every 24 h.

### Bioinformatics Analyses

GAPDH of *L. monocytogenes* (GAPDH-LM) similarity searches were done online using FASTA (available at http://www.ebi.ac.uk/fasta33/) and BLAST (available at http://www.ebi.ac.uk/blast2/ and (http://www.ncbi.nlm.nih.gov/sutils/genom_table.cgi). The analysis of protein domains was based on the Pfam database (available at: https://www.ebi.ac.uk/interpro/) (44). Theoretical 3D predictive models for *L. monocytogenes* GAPDH (GAPDH-LM), *M. tuberculosis* GAPDH (GAPDH-MTB), and *S. pyogenes* GAPDH (GAPDH-SP) were obtained using the Automated Comparative Protein Modeling Server SWISSMODEL (available at https://swissmodel.expasy.org/). Multiple alignment and phylogenetic trees of GAPDH from *L. monocytogenes, M. tuberculosis, M. marinum, M. chelonae, S. agalactiae, S. pneumoniae*, and *S. pyogenes* were carried out using Clustal Omega, a multiple sequence alignment program that uses seeded guide trees and HMM profile-profile techniques (available at https://www.ebi.ac.uk/Tools/msa/clustalo/). The aligned regions correspond to the InterPro domain IPR020828 that all the proteins have at the beginning of their sequence. The InterPro domain IRP020828 corresponds to the glyceraldehyde 3-phosphate dehydrogenase, NAD(P) binding domain: https//www.ebi.ac.uk/interpro/entry/InterPro/IPR020828/. The consensus symbols of the alignments were taken from https://www.ebi.ac.uk/seqdb/confluence/display/JDSAT/Clustal+Omega+FAQ#ClustalOmegaFAQ-Whatdotheconsensussymbolsmeaninthealignment? Their meaning is the following: an ^*^ (asterisk) indicates positions which have a single, fully conserved residue; a: (colon) indicates conservation between groups of strongly similar properties as below—roughly equivalent to scoring >0.5 in the Gonnet PAM 250 matrix: (STA, NEQK, NHQK, NDEQ, QHRK, MILV, MILF, HY, FYW); a. (period) indicates conservation between groups of weakly similar properties as below—roughly equivalent to scoring = <0.5 and >0 in the Gonnet PAM 250 matrix (CSA, ATV, SAG, STNK, STPA, SGND, SNDEQK, NDEQHK, NEQHRK, FVLIM, HFY). Note that TV is included in the weaker scoring groups despite scoring 0–0 in the PAM 250 matrix, this is because it is a fairly common substitution as they are both beta-branched in fully buried residues, at the cost of a hydrogen bond. In fact, this substitution has been used in the past to make TS mutants (Information courtesy of Toby Gibson).

### cDNA Plasmids, *in vitro* Transcription and Recombinant Proteins

cDNA plasmid clones of antigens from *L. monocytogenes* serovar 1/2 (listeriolysin O, LLO, and glyceraldehyde-3-phosphate-dehydrogenase, GAPDH), Ag85A antigen of *M. marinum*, or pneumolysin from *S. pneumoniae* were obtained from Bioclone Inc. Plasmids were first linearized to prepare mRNA by *in vitro* transcription (Qiagen *in vitro* transcription kit) and mRNA transcripts purified with spin columns that contain a silica-based membrane. Purity and concentrations were measured by Nanodrop and further quantification of purity and the size of transcripts was verified by electrophoresis. *Escherichia coli* strain BL21 bearing plasmids to express large quantities of His-fusion recombinant full-length proteins of LLO (LM-LLOrec or LLOrec) and GAPDH of *L. monocytogenes* (LM-GAPDHrec or GAPDHrec), pneumolysin O (PLYrec) of *S. pneumoniae*, and Ag85A of *M. marinum* (Ag85Arec) were obtained from Bioclone Inc. The expression of large quantities His-fusion proteins was induced with 1 mM IPTG for 5 h at 37°C. His-tagged recombinant proteins were purified with TALON resin, according to the manufacturer's instructions (Clontech). Purification of recombinant proteins was evaluated after SDS-PAGE gels loading 3 μg of protein per lane and Coomasie staining (**Figure 2A**, labeled as His-protein expression in *E. coli*) as previously reported by our group (39). Verification of protein purification was evaluated after cutting the bands from gels, TCA precipitation, and proteomic identification at the Centro Nacional of Biotechnology (Madrid). Protein purification was passed through the ToxinEraserTM kit (Genescript, catalog number L0038) to eliminate traces of endotoxin recombinant purified proteins and traces of endotoxin verified with the Genescript ToxiSensorTM chromogenic Limulus Amebocyte lysate kit (catalog number L0035C). The endotoxin elimination kit consists of columns composed by an affinity matrix of modified polymyxin B. Endotoxin levels in protein purifications were lower than 0.1 EU/mL, according to the manufacturer. All reagents to be incubated with DC were tested for endotoxin traces and confirmed to have <0.1 EU/mL of endotoxin.

### Preparation of mRNA Encoding Antigens Conjugated to Lipid Carriers (mRNA-Antigen-LIPO)

We prepared the lipid carriers using lipofectamine 2000 (Invitrogen) (5 μl) which was added to mRNA encoding antigens (GAPDH, LLO, PLY and Ag85A) prepared in the previous section before (100 pmol), in a total volume of 100 μL of Opti-MEM. mRNA encoding antigens and lipofectamine mixtures (mRNA-antigens-LIPO) were incubated for 1 h at RT to allow conjugation to mRNA, followed by 5 min of incubation in a water-bath sonicator to allow for the forming of liposome-like carriers. DC prepared in 6-well-plates (1 × 10^6^/well) were incubated with mRNA encoding antigens-LIPO mixtures in Opti-MEM medium without serum for 4 h. Supernatants were removed and cells were incubated for 12 h in DMEM-1% FCS. Efficiencies of mRNA uptake by DC are shown in **Figure 2A** (DC lysates Coomasie gel of immunoprecipates). Briefly, DC were loaded with 50 μg/mL of mRNA encoded PLY, Ag85A, LLO, or GAPDH conjugated to the lipid carrier, lipofectamine for 16 h. Next, DC were lysed and immunoprecipitated with rabbit anti-Mycobacterium antibody (Colorado University), rabbit anti-PLY (a gift of JR de los Toyos, Oviedo, Spain), and rabbit anti-Listeria monocytogenes GAPDH1-22 antibody (performed by C. Alvarez-Dominguez and M. Fresno at CBMSO facilities using GAPDH1-22 peptide and incomplete Freund's adjuvant) as previously reported (24). Immunoprecipates were stained with Coomasie blue.

### Preparation of Murine DC Vaccines and Assays for DC Activation

Bone-marrow derived DC cells obtained from mice femurs were differentiated with GM-CSF (20 ng/mL) for 7 days. Differentiated DC presented a phenotype of 98% CD11c^+^MHC^−^II^+^CD11b^−^/^+^CD40^−^CD86^−^ cells. These DC were used *in vivo* for T cell responses or vaccination protocols. For DC activation assays, differentiated DC were treated with different reagents for 16 h: 5 μg/mL of recombinant proteins LM-GAPDHrec or LM-LLOrec or 50 μg/mL of mRNA-LIPO complexes: mRNA-LLO-LIPO and mRNA-GAPDH-LIPO. Two adjuvants were also included as reference controls: LPS (10 ng/mL) and the Th1 adjuvant DIO-1 (10 ng/mL). Cell surface markers of DC activation were explored by flow cytometry. Activated DC presented a phenotype of 90% CD11c^+^IAb^+^CD40^+^CD86^+^ positive cells. Activation was also measured in DC supernatants after filtration and storage at −80°C to measure cytokine production using a multiparametric CBA kit of BD Biosciences (see Cytokine Measurement section).

### Cell Toxicity and Apoptosis Assays on Macrophages and DC Vaccines

Bone-marrow derived macrophages (BM-DM) were obtained, as described above, from mice femurs and differentiated with M-CSF (20 ng/mL) for 7 days. BM-DM and activated DC were treated, or not, with the different recombinant proteins or mRNA encoded antigens conjugated to lipid carriers (50 μg/mL) for 16 h in culture medium, washed, and analyzed for cell toxicity or apoptosis. Cell toxicity was examined with Trypan-blue staining by light microscopy as well as by hemolysis of sheep red blood cells. Apoptosis was examined by flow cytometry using two reported products, annexin-V conjugated to allophycocyanin (APC) fluorochrome and 7-AAD (7-aminoactinomycin D) (BD Biosciences, San Jose, CA, USA). Staining of cells with 7-ADD corresponded to necrotic cell death, whereas staining of cells with annexin-V alone corresponded to apoptotic programmed cell death (mean ± SD). Results are expressed as the % of cell toxicity or as the percentages of apoptotic cells ± SD of triplicate samples, respectively (*P* < 0.05).

### Virulence of Bacterial Clinical Isolates *in vitro* and *in vivo*

**Figure 1:**
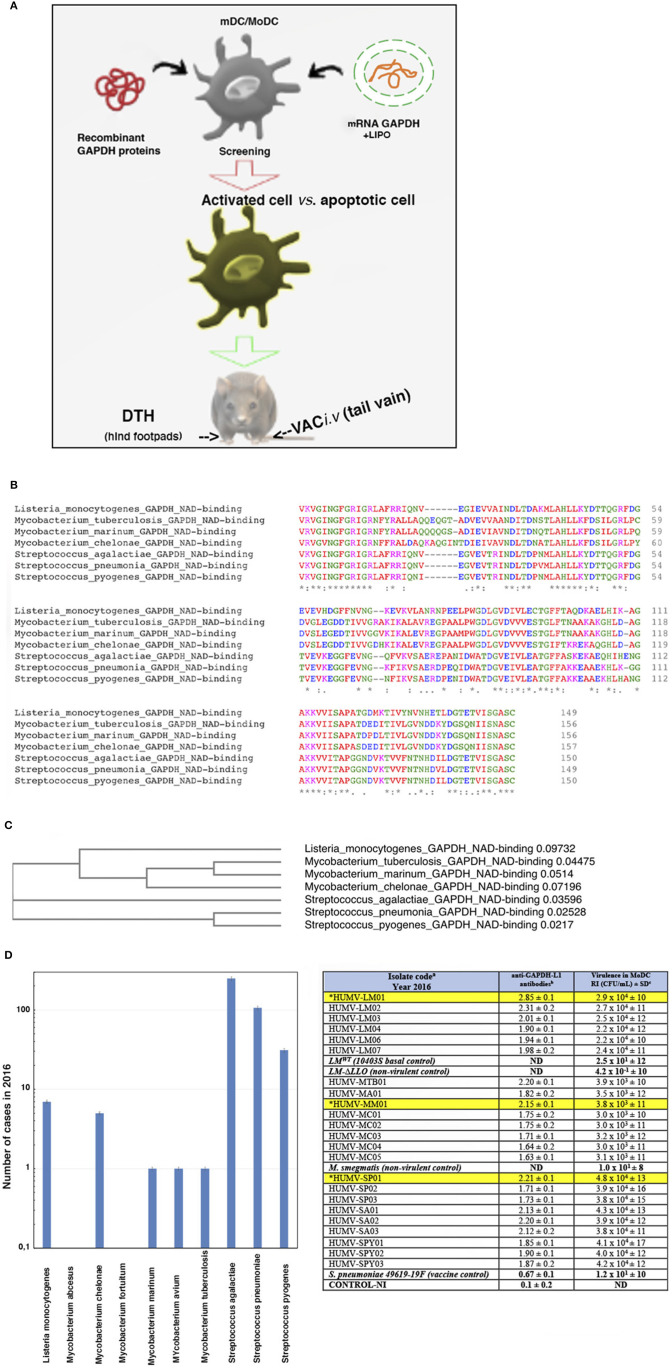
Scheme of the study and selection of the bacterial antigens for the vaccine vectors. **(A)** Scheme explaining our strategy in this study. First DC are incubated with the different antigen forms: recombinant *L. monocytogenes* GAPDH proteins or mRNA-LIPO-GAPDH carriers for screening of the suitable ones, causing DC activation with minimal apoptosis induction. DC vaccines loaded with the different antigens are tested for DTH responses as a measurement of T cell responses. DC vaccines with the maximal DTH responses are tested for vaccination experiments followed by bacterial challenge. **(B)** Multiple alignment of GAPDH sequences of NAD-binding domains of the following bacteria detected at our Health Institution showing more than 95% homology: *Listeria monocytogenes* (A0A0B8RGN3_LISM), *M. tuberculosis* (A0A045ITJ4_MYCTX), *M. chelonae* (A0A0E3TR96_MYCCH) *M. marinum* (A0A2Z5YDP2_MYCMR), *S. agalactiae* (Q9ALW2_STRAG), *S. pyogenes* (G3P_STRPY), and *S. pneumoniae* (I6L8L9_STREE) protein sequences using CLUSTAL O (1.2.4) multiple sequence alignment. The aligned regions correspond to the InterPro domain IPR020828 that all the proteins have at the beginning of their sequence. The InterPro domain IPR020828 corresponds to the Glyceraldehyde 3-phosphate dehydrogenase, NAD(P) binding domain: https://www.ebi.ac.uk/interpro/entry/InterPro/IPR020828/. The consensus symbols are taken from https://www.ebi.ac.uk/seqdb/confluence/display/JDSAT/Clustal+Omega+FAQ#ClustalOmegaFAQ-Whatdotheconsensussymbolsmeaninthealignment? The symbols meaning is explained in *Materials and Methods section Bioinformatics Analyses*. Colors on protein alignments correspond to residues according to their physicochemical properties: RED corresponds to Small (small ^+^ hydrophobic-including aromatic-Y), BLUE corresponds to acidic, MAGENTA corresponds to basic—H, GREEN corresponds to hydroxyl ^+^ sulfhydryl ^+^ amine ^+^ G and GRAY corresponds to unusual amino/imino acids (see Supplementary Material for complete residues description). **(C)** Phylogenetic tree of the seven bacteria species compared in this study. The tree data are the following: (Listeria_monocytogenes_GAPDH_NAD-binding:0.09732, ((Mycobacterium_tuberculosis_GAPDH_NAD-binding:0.04475, Mycobacterium_marinum_GAPDH_NAD-binding:0.05140):0.03060, Mycobacterium_chelonae_GAPDH_NAD-binding:0.07196):0.31226):0.14525, Streptococcus_agalactiae_GAPDH_NAD-binding:0.03596, (Streptococcus_pneumonia_GAPDH_NAD-binding:0.02528, Streptococcus_pyogenes_GAPDH_NAD-binding:0.02170):0.01751); **(D)** Analyses of the clinical cases of bacteria species after the bioinformatic analysis of GAPDH sequences showing 95% homologies in *B* and detected in the year 2016 at the Hospital U. Marqués de Valdecilla (Microbiology Dpt) from a complete study from 2014 to 2019 (graphic on the left). In the Table (on the right), we show sera from patients (HUMV codes) infected with the bacterial strains of *B* and examined for anti-LM-GAPDH_1−22_ antibodies using a peptide ELISA. Sera were collected from patients and storage at −80°C. In the table, we present the antibody titers of patients from a representative 2016 year and with anti-GAPDH-L1 titers higher than 2.0 OD after performing a peptide-specific ELISA. Results are presented as the mean ± SD of OD units in triplicate experiments (*P* < 0.05). Asterisks and highlighted in yellow correspond to the selected clinical isolates for our further study. Virulence of these clinical isolates into human MoDC (2 × 10^5^/mL) from healthy donors is evaluated *in vitro* after MoDC infection with 2 × 10^6^ CFU/sample of the clinical isolates detailed in the Table. MoDC were lysed at two different times, at 1 h and at 16 h infection and lysates cultured in agar plates to count CFU. *In vitro* virulence is expressed as a replication index (RI) of the ration of CFU at 16 h to CFU at 1-h post-infection. Results expressed as RI numbers ± SD of three different experiments. ELISA and virulence assays *in vitro* using MoDC are performed in triplicate. A Student *t*-Test is applied for statistical analysis (*P* ≤ 0.05).

DC vaccines prepared in mice (1 × 10^6^ cells/mL) were infected at a MOI of 10:1 (bacteria: cells) to evaluate the *in vitro* replication of invasive clinical isolates of LM (HUMV-LM01), MM (HUMV-MM01), and SP (HUMV-SP01) which were calculated as replication indexes (RI) as previously reported (2, 27, 39). RI are calculated by the CFU at 16 h post-infection, divided into CFU at 1 h post-infection. This parameter is considered an indicator of bacterial growth in DC and is comparable to *in vivo* virulence in spleens 72 h post-infection, as we have previously reported for listeriosis (27). We included the following bacteria as controls: LM 10403S strain (LMWT) as the LM basal control, LLO deficient strain, LMΔLLO as non-pathogenic LM, Mycobacterium smegmatis as non-pathogenic mycobacteria control and the vaccine strain 49619-19F of *S. pneumoniae* as the non-pathogenic SP control (Figure 1C). Similarly, to evaluate virulence *in vivo*, we inoculated intravenously 104 CFU of each clinical isolate to be tested. 104 CFU/mice corresponded to a bacterial dose lower than LD50 (see section Mice for LD50 calculations). Spleen homogenates were plated in agar plates to count CFU and results are expressed as CFU/mL. Bacterial controls were the same as those used for *in vitro* virulence assays.

### Delayed Type Hypersensitivity (DTH) Reactions Elicited by DC-Vaccines

C57BL/6 mice were immunized *i.p* with LM (HUMV-LM01), MM (HUMV-MM01), or SP (HUMV-SP01) (5 × 10^3^ CFU). Seven days later, mice were inoculated in the left hind footpads using DC vaccines (10^6^ cells/mice) pre-loaded with the following reagents: the recombinant proteins of *L. monocytogenes* LLO_rec_, and GAPDH_rec_, *M. marinum* Ag85A_rec_ or *S. pneumoniae* PLY_rec_, or the mRNA-Ag-LIPO complexes: mRNA-LLO-LIPO, mRNA-GAPDH-LIPO, mRNA-Ag85A-LIPO, or mRNA-PLY-LIPO. DC vaccines were formulated in the presence of DIO-1 (2 μg/mL) (2). The negative controls were the right hind footpads, since they were not inoculated. After 48 h, we measured the footpad thickness with a caliper; results are expressed in millimeters as the mean of three different experiments. To explore T cell responses in detail, we collected and homogenized the popliteal lymph nodes of mice analyzed for DTH reactions and cell homogenates were passed through cell strainers to analyze CD4^+^ and CD8^+^ T cells by flow cytometry. Results are expressed as the percentage of positive cells ± SD.

### Vaccination Experiments With DC Vaccines Loaded With *Listeria* Recombinant Proteins or mRNA-LIPO

C57BL/6 female mice were vaccinated (*n* = 5/vaccine), or not (*n* = 5), *via* the lateral tail vain (*i.v*), with one dose of DC-vaccines (10^6^ cells/mice) pre-loaded with recombinant *Listeria* proteins as GAPDH_rec_ and LLO_rec_; mRNA-LIPO complexes such as mRNA-LIPO-LLO and mRNA- LIPO-GAPD-; or empty DC-LIPO. Seven days post-vaccination, mice were challenged *i.v* with 100 μL bacterial suspension of LM (HUMV-LM01), MM (HUMV-MM01), or SP (HUMV-SP01) in saline (1 × 10^4^ CFU/mL). All animals were examined daily and 14 days after the bacterial challenge, the mice were bled and sacrificed to quantify viable CFU/mL in the spleens and the cytokines in mice serum. Results are expressed as the percentages of protection ± SD of CFU/mL of vaccinated vs. non-vaccinated animals, using two controls: empty DC and saline. CFU of non-vaccinated mice are as follows: saline LM (HUMV-LM01) 2.75 × 10^5^ CFU/mL, DC-CONT LM (HUMV-LM01) 2.60 × 10^5^ CFU/mL, saline MM (HUMV-MM01) 1 × 10^5^ CFU/mL, DC-CONT MM (HUMV-MM01) 0.9 × 10^5^ CFU/mL, saline SP (HUMV-SP01) 2.5 × 10^5^ CFU/mL, and DC-CONT SP (HUMV-SP01) 2.49 × 10^5^ CFU/mL.

### Intracellular IFN-γ Staining

Spleen cells of vaccinated and non-vaccinated mice were cultured in 96-well plates (5 × 10^6^ cells/mL) and stimulated with *L. monocytogenes* GAPDH_1−22_ peptide (50 μM) for 5 h in the presence of brefeldin A. Cells were surface labeled for CD4 or CD8, fixed, and permeabilized with a cytofix/cytoperm kit to measure IFN-γ (BD Biosciences). After sample acquisition by flow cytometry, data were gated for CD4^+^ or CD8^+^ events, and the percentages of these cells expressing IFN-γ were determined. Results were corrected according to the percentages of total CD4^+^ or CD8^+^ positive cells. Data were analyzed using FlowJo software (Treestar, Ashland, OR, USA).

### Peptide-ELISA Assay to Measure *Listeria monocytogenes* GAPDH_1−22_ Antibody Titers

Ninety-six –well-plates were coated with *L. monocytogenes* GAPDH_1−22_ peptide (50 μg/mL) and coated to 96-well-plates in carbonate buffer (pH 8.0) overnight at 4°C. Plates were washed and incubated with 1 mg/mL of BSA (fraction V) to saturate all sites in the plates. Sera of patients infected with LM, MM, or SP or sera of vaccinated or non-vaccinated mice were 1/10 diluted and peptide coated plates were incubated with diluted sera for 2 h at RT, as previously described (2, 24). Reactions were developed with goat anti-human IgG or goat anti-mouse IgG and absorbances were analyzed at 450 nm. Results are presented as optical density measurements (OD) from mean values ± SD, of triplicate experiments.

### Isolation of MoDC From Healthy Donors and *in vitro* Virulence With Clinical Isolates

Leukocytes from whole blood cells were isolated as the interphase of a Ficoll gradient. Leukocytes were incubated with microbeads conjugated to a mouse IgG2a monoclonal anti-CD14 antibody, and passed through MACS™ columns (Miltenyi, Bergisch Gladbach, Germany) to select monocytes (Mo) as CD14^+^ positive cells. Mo cells were differentiated to monocyte derived DC (MoDC) using standard procedures previously reported (27). In brief, Mo (1 × 10^6^ of cells/mL) are cultured into 6-well-plates (Falcon™) over 7 days using GM-CSF (50 ng/mL) and IL-4 (20 ng/mL) in RPMI-20% FCS medium. Differentiated cells were 98% CD45^+^HLA-DR^±^CD86^−^CD14^−^ positive cells and were used for the *in vitro* virulence analysis.

### Adjuvant Effects of Vaccine Vectors on to MoDC From Healthy Donors

MoDC (2 × 10^6^ cells/mL) were incubated with different recombinant proteins (5 μg/mL) or adjuvants (20 ng/mL), LLO_rec_, GAPDH_rec_, LPS, or DIO-1. After 16 h, supernatants were collected, filtered, and stored at −80°C until use for the cytokine analysis. Cell surface markers were analyzed by flow cytometry to evaluate the percentages of CD45, MHC-II, CD86, and CD14 positive cells to determine an activation phenotype of 99% CD45^+^HLA-DR^++^CD40^++^CD86^++^ positive cells.

### Cytokine Measurement

Cytokines in mice sera, DC, or MoDC supernatants were quantified using multiparametric CBA kits, either for mice or for human samples (BD Biosciences, San Jose, CA, USA). The human Th1/Th2/Th17 CBA kit (catalog number 560484) was used to measure human cytokines in MoDC supernatants, and the mouse Th1/Th2/Th17 CBA kit (catalog number 560485) was used to measure cytokines in mice sera and DC supernatants. Cytokine concentrations were expressed as the average of three replicates in pg/mL ± SD. ANOVA was applied to these samples according to the manufacturer's instructions. Data were analyzed using the FlowJo software.

### FACS Analysis

Cell surface markers of human MoDC, murine DC, or murine spleens were analyzed by FACS using the following antibodies: anti-HLA-DR-FITC, anti-CD45-PerCP, anti-CD14-PE, and anti-CD86-V450 (clone 2331) for human MoDC. For cell surface markers of murine DC, we used, biotin anti-IAb (clone AF6-120-1), anti-CD11c-PE (clone HL3), anti-CD40-APC (monoclonal 3/23 from BD Pharmingen), and anti-CD86-V450 (clone GL-1) and for murine spleens we also used anti-CD4-FITC (clone RPA-T4) and anti-CD8-PE (clone RPA-T8) (BD Biosciences). Data were analyzed using the FlowJo software. ANOVA was applied to these samples according to the manufacturer's instructions.

### Statistical Analysis

For statistical analysis, the Student's *t*-test was applied to mice assays infected with bacterial pathogens. For statistical purposes, each group included five mice for all assays reported (*P* < 0.5 was considered significant). ANOVA analysis was applied to the cytokine measurements and flow cytometry analysis as per the manufacturer's recommendations (*P* ≤ 0.05 was considered significant). For statistical purposes, each flow cytometry sample was performed in triplicate. GraphPad software was used for generation of all the graphs presented.

### Ethics Statement

This study was carried out in accordance with the Guide for the Care and Use of Laboratory Animals of the Spanish Ministry of Science, Research and Innovation. The Committee on the Ethics of Animal Experiments of the University of Cantabria approved the protocol (Permit Number: PI-01-17) that follows the Spanish legislation (RD 1201/2005). All surgeries were performed by cervical dislocation, and all efforts were made to minimize suffering. Similarly, for the use of human data of bacteria clinical isolates, we have an approved project from the Committee of Clinical Ethics of Cantabria (CEm) entitled: “Clinical Development of *Listeria* based vaccines” which includes Informed Consent and General Project Information documents of patients (Permit Acta Number: 29/2014, internal code: 2014.228).

## Results and Discussion

We initiated this study with the hypothesis that bacterial vaccines for adults can benefit from the discovery of antigens that are able to immunodulate DC and drive a wide spectrum immunity that cross-protects against bacterial infectious diseases of the genera *Listeria, Mycobacterium*, and *Streptococcus*. Vaccines inducing cross-protection immunity has recently been suggested for these taxonomic groups as multivalent vaccines (28). They are differentiated from conventional vaccines as they have the capacity of cross-reactive immune responses. For this reason, here, we refer to them as CRV vaccines to differentiate them from other type of vaccines, such as trained-immunity based vaccines (TIbV). CRV and TIbV might share two features: (i) stimulation of non-specific protection against several pathogens that involves innate immune cells and (ii) induction of specific immune responses to the vaccine antigens (5, 6). DC are innate immune cells responsible for antigen presentation and is relevant in all types of vaccines, conventional, CRV, or TIbV. DC are explored here as vaccine platforms to evaluate any bacterial antigen as a candidate for cross-protection vaccination, if the antigens induced minimal DC apoptosis, along with maximal expansion of T cells. In this context, two types of vaccine carriers are explored: an mRNA-encoded antigen conjugated with lipid carriers and recombinant proteins (see scheme of our procedure in Figure 1A). The bacterial antigens we use in this study are those reported in experimental vaccines for the above-mentioned bacteria genera: *L. monocytogenes* GAPDH and LLO, *M. marinum* Ag85A and *S. pneumoniae* PLY (37–42). DC and macrophages were selected as vaccine vectors since they are innate immune cells that participate actively in cross-protection immunity (5, 6). We focused our study to *L. monocytogenes* GAPDH antigen (Lmo2459) since it presents similar ADP-ribosylating abilities, immunogenic domains, and cross-immune responses in three bacterial genera of our study, *Listeria, Mycobacterium*, and *Streptococcus* (24–26, 28). These features prompted us to hypothesize that *L. monocytogenes* GAPDH was a candidate for CRV vaccines.

### Selection of Antigens and Antigen Forms

We performed two approaches to select the bacterial pathogens for our study: first a bioinformatic analysis we previously reported (28) to search for homologies higher than 80% among GAPDH of most common pathogenic bacteria communicated annually at our Health institution and virulence analysis of clinical isolates. From a 5-year study from 2014 to 2018, we chose year 2016 as representative and detected several bacterial genera with GAPDH homologies higher than 80%, such as *Hemophilus, Klebsiella, Listeria, Mycobacterium, Pseudomonas, Staphylococcus*, and *Streptococcus* (Table 1). However, if we quoted GAPDH homologies to 95% or higher, only clinical isolates of the bacterial taxonomic groups of *L. monocytogenes, Mycobacterium*, or *Streptococcus* fitted this category. The highest GAPDH homologies corresponded to *L. monocytogenes* (LM), *M. chelonae* (MC), *M. tuberculosis* (MTB), *M. marinum* (MM), *S. agalactiae* (SA), *S. pneumoniae* (SP), and *S. pyogenes* (SPY). Next, we focused on the NAD-binding domains of GAPDH from these taxonomic groups, using CLUSTAL 0 (1.24) multiple sequence alignment (Figure 1B), and, observed that protein sequences covering amino acids 3–25 displayed the highest identities (asterisks corresponds to 100% identity, colon symbol to 90%, and period symbol to 80% (*detailed analysis is described in Material and Methods, section Bioinformatics Analyses*). Amino acids are shown in a colored codes to distinguish homologies (Figure 1B) and color code explanations are provided in the Figure legend and Supplementary Material (Supplementary Table 1). These alignments might explain that the peptide-specific anti-*Listeria monocytogenes* GAPDH_1−22_ antibody prepared in rabbits, with the 1-22 amino acid sequence of LM, can also detect MTB, MM, and SP bacterial extracts and surface shapes of the bacteria as previously described by our group (24, 28), suggesting that LM, MTB, MM, and SP shared immunogenic domains, in addition to enzymatic abilities and enzymatic domains. This is especially relevant as the phylogenetic tree relates NAD-binding domains of LM with MTB, MM, and MC. Another branch of the phylogenetic tree relates the NAD-binding domains of LM and SA and a third branch relates NAD-binding domains of LM with SP and SPY (Figure 1C), suggesting that GAPDH could be a common virulence factor. To further investigate this issue, we collected sera from all patients reported with infections caused by these eight bacterial species, detected at year 2016 (graph plot in Figure 1D), and explored for the presence of antibodies recognizing the LM-GAPDH_1−22_ peptide, using a peptide-ELISA previously described (27). Several patients with infections caused by LM (HUMV-LM01, HUMV-LM02, and HUMV-LM03), MM (HUMV-MM01), MTB (HUMV-MTB01), SP(HUMV-SP01), SA (HUMV-SA01, HUMV-SA02, HUMV-SA03), and SPY (HUMV-SPY02) presented very high levels of antibodies recognizing the LM-GAPDH_1−22_ epitope with O.D. ≥ 2.0, (right table in Figure 1D, column labeled as anti-GAPDH-L1 antibodies), while the remaining patients presented high levels of antibodies with O.D. ≥ 1.5. We concluded that immune responses generated by *Listeria, Mycobacterium*, and *Streptococcus* taxonomic groups are mainly targeted to a common GAPDH_1−22_ epitope, strongly suggesting that GAPDH might be a common virulence factor to these pathogens. Evaluation of the *in vitro* virulence of their clinical isolates also supports our hypothesis. *In vitro* virulence was performed, infecting monocyte derived dendritic cells (MoDC) from healthy donors with the clinical isolates at a MOI of 10:1 and examining the bacteria replication indexes (RI). RI are defined as the ratio of CFU/mL at 16 h post-infection to CFU/mL at 1 h (27). We detected that those patients with the highest titers of antibodies recognizing the LM-GAPDH_1−22_ epitope, also presented the highest virulent strains of LM, MTB, MM, SP, or SPY, showing at least 100-fold higher replication indexes (RI) than non-virulent strains (right table in Figure 1D, column labeled as virulence in MoDC). This methodology was confirmed by the virulence of the clinical isolates *in vivo* using C57BL/6 mice and bacterial doses lower than LD50 [see Material and Methods section Mice and (41–44)], that reported similar results as *in vitro* virulence using MoDC (Supplementary Table 2). In brief, these data strongly suggests that GAPDH is a common virulence antigen of *Listeria, Mycobacterium*, and *Streptococcus* that needs to be explored as a candidate for CRV vaccines.

**Table 1:** Main bacteria detected at Hospital Universitario Marqués de Valdecilla yearly with GAPDH showing more than 80% sequence homology at N-terminus.

**^a^Bacteria**	**N^**°**^ isolates**
^*^Escherichia coli	3,748
*Haemophilus influenzae*	122
*Haemophilus parainfluenzae*	36
*Klebsiella pneumoniae*	766
*Listeria monocytogenes*	7
*Mycobacterium avium complex*	1
*Mycobacterium avium*	1
*Mycobacterium chelonae*	5
*Mycobacterium marinum*	1
*Mycobacterium tuberculosis complex*	14
*Mycobacterium tuberculosis*	1
*Pseudomonas aeruginosa*	746
*Staphylococcus aureus*	1,012
*Staphylococcus epidermidis*	363
*Streptococcus agalactiae*	248
*Streptococcus pneumoniae*	106
*Streptococcus pyogenes*	31

a *Bacteria genera and species with GAPDH sequence homology higher than 80% detected in clinical isolates of the Microbiology Department at HUMV in the year 2016, a median time of the 5-year analysis we have performed*.

* *Escherichia coli was included as negative control because GAPDH sequence homology was 60% and because it is the most abundant infection at HUMV*.

**Figure 2:**
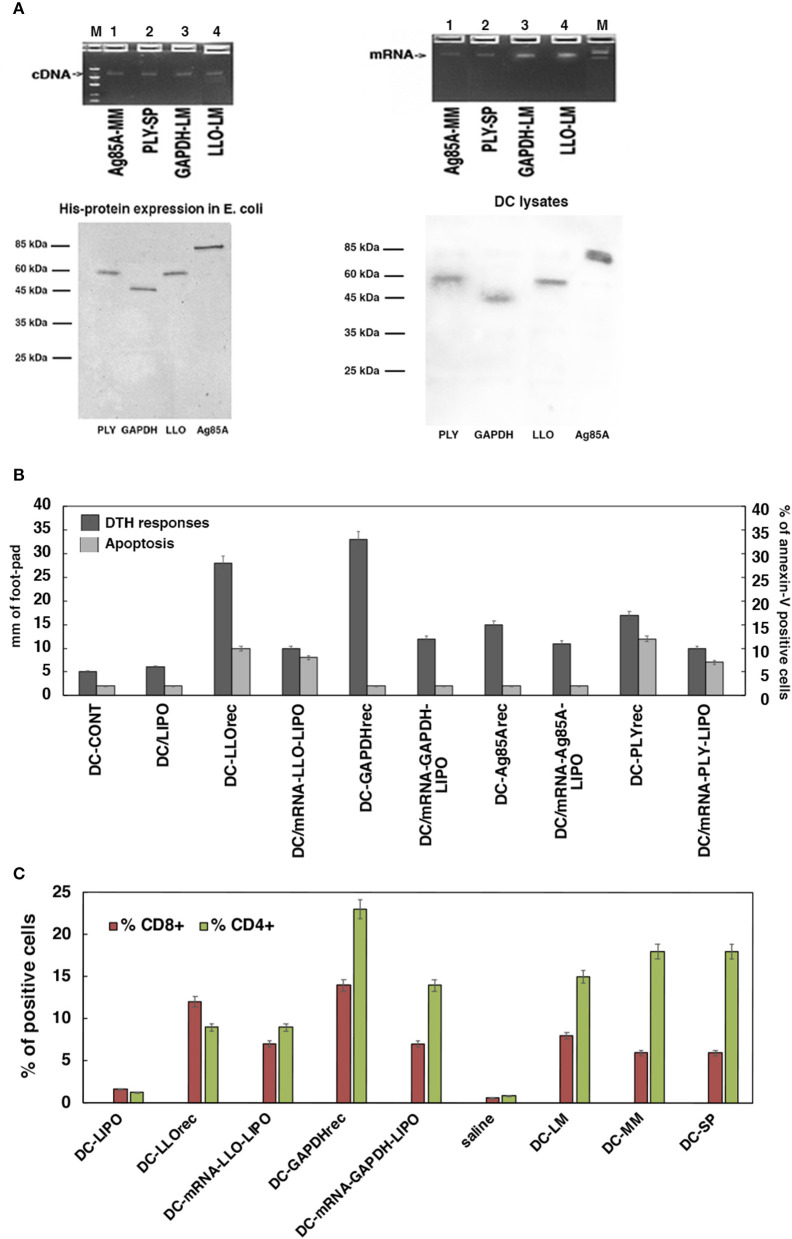
Selection of the antigen forms for the vaccines. **(A)** Upper geles correspond to preparation of the mRNA antigens from cDNA plasmids encoding for Ag85A antigen of *M. marinum* (MM), PLY of *S. pneumoniae* (SP), and GAPDH or LLO of *L. monocytogenes* (LM) after *in vitro* transcription. Gels show the linear plasmids (upper left) and mRNA transcripts (upper right). Concentration of the mRNA preparations and qualities are shown in Supplementary Figure 1A. Lower gels correspond to Coomasie stained gels of purification of His-recombinant proteins (lower left) and DC uptake of prepared mRNA from all antigens (PLY of SP, Ag85A of MM, LLO, or GAPDH of LM) conjugated to the lipid carrier, lipofectamine, and after 16 h DC cells are lysed. Lysates were immunoprecipitated with rabbit anti-LLO antibody (DIATEVA), rabbit anti-Mycobacterium antibody (Colorado University), rabbit anti-PLY (a gift from JR de los Toyos, Oviedo, Spain), and rabbit anti-LM-GAPDH1-22 antibody (C. A-D and M.F obtained at CBMSO) (24). All immunoprecitpates were stained with Coomasie blue. **(B)** DC apoptosis (light gray bars) and DTH responses measured as the footpad swelling (dark gray bars) evaluated after incubation of DC with different antigens: empty DC (DC-CONT), lipofectamine (DC-LIPO), recombinant proteins as PLYrec from SP, LLOrec, and GAPDHrec from LM and antigen Ag85A from MM or mRNA-LIPO complexes of PLY, LLO, GAPDH, or Ag85A. Apoptosis is measured *in vitro* by flow cytometry and results are expressed as the percentages of annexin-V positive cells ± SD of three different experiments. ANOVA test was applied for flow cytometry results (*P* ≤ 0.05). DTH responses are measured *in vivo* after inoculation of right hind footpads of C57BL/6 mice with the different DC vaccines (*n* = 5 per DC vaccine). Forty-eight hours after inoculation of DC vaccines, DTH responses are evaluated by the swelling of the hind footpads measured with a caliper. Results are expressed as millimeters ± SD of each group of 5 mice. Student *t*-Test was applied for statistical analysis (*P* ≤ 0.05). **(C)** C57BL/6 mice were immunized i.v with 5 × 10^3^ CFU/mice (HUMV-LM01, HUMV-MM01, or HUMV-SP01) and 7 days later, left hind footpads were inoculated with 1 × 10^6^ DC vaccines (pre-loaded with 5 μg/mL of LLOrec, GAPDHrec, or 50 g/mL of mRNA-LIPO-GAPDH or mRNA-LIPO-LLO, or 1 × 10^6^ CFU of LM, MM or SP, LIPO incubated DC, or saline incubated DC). Popliteal lymph nodes are isolated from mice legs and after homogenization, T cells sub-populations are analyzed by flow cytometry. Percentages of CD4^+^ (green bars) or CD8^+^ T cells (red bars) are shown. Results are expressed as the percentages of positive cells ± SD of three different experiments. Student *t*-Test was applied for statistical analysis (*P* < 0.05).

The second approach was to decipher the best antigen form to prepare a T-cell based vaccine vector from using DC loaded with the antigens and inoculation of mice hind footpads to examine a classical delayed-type hypersensitivity assay (DTH), a valid measure of T cell immunity (2). The antigens included in this strategy are commercially available as cDNAs (Bioclone Inc): Ag85A of MM (Ag85-MM), pneumolysin (PLY) of SP (PLY-SP), and GAPDH (GAPDH-LM) and listeriolysin O (LLO) of LM (LLO-LM). We prepared and compared two types of antigen forms, recombinant proteins and mRNA-lipid carrier complexes (mRNA-LIPO) because they can load different antigen processing compartments on DC. While recombinant proteins load the endo-lysosomal compartments relevant for MHC-class II antigen presentation, mRNA-lipid carrier complexes (LIPO) load the cross-presentation compartments relevant for MHC-class I antigen presentation (30, 45, 46). To prepare mRNA-lipid carrier complexes, commercially available DNA plasmids were first linearized (left upper cDNA gel in Figure 2A showed cDNA plasmids of each antigen) and mRNA samples were obtained by *in vitro* transcription (right upper mRNA gel in Figure 2A). Next, we added a CAP site at the 5′ end and a poly A tail at the 3′ end, following the manufacture's recommendation (see details in Materials and Methods, section cDNA Plasmids, *in vitro* Transcription, and Recombinant Proteins) (concentration and purity of transcripts are shown in Supplementary Figure 1A). Next, mRNA samples (100 pmol) were incubated with the lipid carrier, lipofectamine (5 μL), to obtain mRNA-Antigen-lipid carrier complexes (labeled here as mRNA-antigen-LIPO) and offered to DC to evaluate maximal uptake by antigen presenting cells (right lower Coomasie stained gel in Figure 2A). To prepare recombinant proteins, commercially available DNA plasmids were expressed in large quantities as His-fusion proteins in *E. coli* strain BL21 to obtain LLO_rec_, Ag85A_rec_, PLY_rec_, or GAPDH_rec_ (left lower Coomasie stained gel in Figure 2A). Toxicities of mRNA-antigen-LIPO complexes and recombinant proteins were examined by hemolysis of sheep red blood cells in macrophages (BM-DM) and DC (Supplementary Figure 1B), as well as by Trypan blue staining in DC which reflects cell viabilities (Supplementary Figure 1C). Both methods of analyzing toxicities—hemolysis and Trypan blue—are relevant when using cytolysins (LLO or PLY) that are able to lyse red blood cells as LLO or PLY, while not causing significant reductions on cell viabilities. In fact, the high hemolysis detected with both cytolysins in macrophages, either as recombinant proteins or mRNA-antigen-LIPO complexes (Supplementary Figure 1B), drove us not to use macrophages for vaccine platforms. None of the antigen forms we used with DC caused hemolysis (Supplementary Figure 1B) or reduction of cell viability (Supplementary Figure 1C), therefore, we concluded that DC were the most suitable vaccine platform.

### Selection of Most Immunogenic Antigens

Once antigen forms were prepared, we examined the DTH responses in C57BL/6 mice previously challenged intravenously (*i.v*) with the pathogens LM (HUMV-LM01), MM (HUMV-MM01), or SP (HUMV-SP01). Seven days post-infection we inoculated the left hind footpads with 10^6^ DC pre-loaded with the different bacterial antigens, either recombinant proteins (5 μg/mL) or mRNA-LIPO complexes (50 μg/mL), in solutions with DIO-1 adjuvant to amplify the immune response, per mouse. The DTH response was measured as the swelling on the left hind footpad of each mouse 48 h post-inoculation, compared to the right hind footpad, which acts as the negative control. DC loaded with recombinant LM-GAPDH_rec_ presented the highest DTH responses, followed by DC loaded with recombinant LM-LLO_rec_, next were mRNA-LM-GAPDH-LIPO and mRNA-SP-PLY-LIPO complexes. DC loaded with MM-Ag85A_rec_, mRNA- MM-Ag85A-LIPO complexes (dark gray bars in Figure 2B) induced significant DTH responses but lower than LM-GAPDH or LM-LLO antigen forms. DC loaded with SP-PLY_rec_ and mRNA-SP-PLY-LIPO show half the footpad swelling than GAPDH antigen forms, therefore they induce only partial DTH responses. We also explored the abilities of these antigens to induce apoptosis in DC as a measure of the undesired inactivation of DC (≥10% apoptosis) (see Material and Methods in section Cell Toxicity and Apoptosis Assays on Macrophages and DC Vaccines). Whole pathogens, LM, MM, or SP (HUMV-LM01, HUMV-MM01, or HUMV-SP01, respectively) induced high levels of apoptosis (12–17%) as well as recombinant cytolysins like SP-PLY_rec_ and LM-LLO_rec_ (11–18%) or mRNA-LIPO complexes of these cytolysins (10–13%) (light gray bars in Figure 2B). All the other molecular forms tested (mRNA-LIPO complexes of LM-GAPDH or MM-Ag85A, and their recombinant proteins) presented apoptosis below 5% and similar to controls: DC loaded with lipofectamine (DC-LIPO) or incubated with saline (DC-CONT) (Figure 2B). Therefore, we concluded that the highest immunogenic and less apoptotic antigen forms corresponded to recombinant LM-GAPDH_rec_. mRNA-LIPO complexes of LM-GAPDH show half the lower immunogenic DTH responses than LM-GAPDH_rec_, although we inoculated a 10-fold concentration of mRNA-LIPO complexes compared to recombinant proteins. In brief, we do not consider this antigen form, mRNA-LIPO complexes, as suitable for exploring CRV vaccines. Next, we collected the popliteal lymph nodes of mice with the highest DTH immune responses (LM-GAPDH_rec_, LM-LLO_rec_, mRNA-LIPO complexes of LM-GAPDH or LM-LLO) and cultured them *in vitro* with 1 μg/mL of each antigen for 72 h, and examined the percentages of T cell populations, both CD4^+^ or CD8^+^ T cells by flow cytometry. We detected the highest percentages of CD4^+^ (23%) and CD8^+^ (14%) T cells in mice inoculated with LM-GAPDH_rec_ (Figure 2C). mRNA-LIPO complexes of GAPDH presented significant percentages of CD4^+^ (15%) T cells, but low percentages of CD8^+^ (7%) T cells. The molecular forms of LLO, presented low percentages of CD4^+^ (9%) T cells but significant percentages of CD8^+^ (12%) T cells. However, mRNA-LIPO complexes of LLO induced low percentages of CD4^+^ (9%) and CD8^+^ (7%) T cells. No significant T cell responses were observed in the controls, DC, or in saline. When we compared these results with the DTH responses, we confirmed a correlation between the highest DTH responses (dark gray bars in Figure 2B) and the highest percentages of CD4^+^ and CD8^+^ T cells induced in the popliteal lymph nodes (Figure 2C). We argue that antigens in vaccine platforms that induced high DTH responses reflect the high expansion of T cell responses they induced and explains their high immunogenicity; both features are specific of the antigen.

### Adjuvant Abilities of Vaccine Vectors

**Figure 3:**
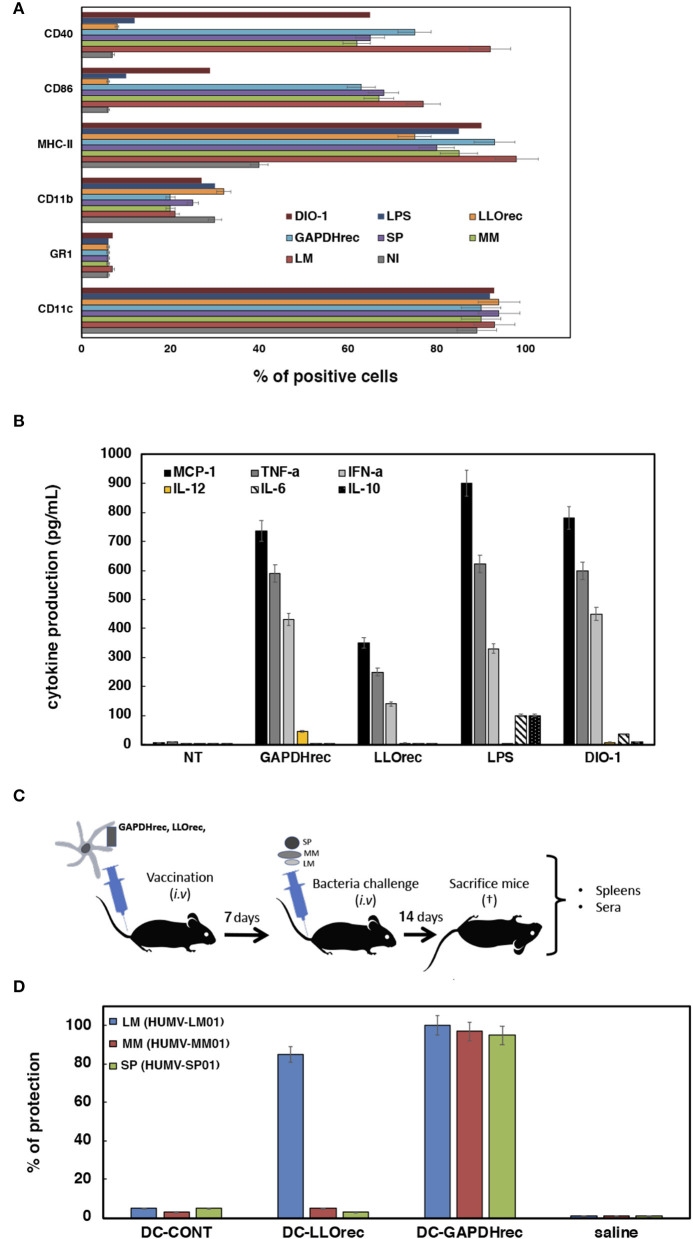
Adjuvant and vaccine abilities of DC vaccine vectors loaded with recombinant or mRNA-LIPO antigens. **(A)** Flow cytometry analysis of DC surface markers after incubation with recombinant proteins LLO_*rec*_ or GAPDH_*rec*_ or mRNA-LIPO complexes: mRNA-LIPO-LLO, mRNA-LIPO-GAPDH, bacteria MM (HUMV-MM01), SP (HUMV-SP01), or LM (HUMV-LM01) or two adjuvants, LPS or DIO-1. Results show the percentages of CD11c^+^, MHC-II^+^, CD40^+^, or CD86^+^ positive cells. Results are the mean of three different experiments ± SD. Student *t*-Test was applied for statistical analysis (*P* ≤ 0.05). **(B)** Cytokine levels released to the supernatants of DC and measured with a multiparametric CBA kit (BD Biosciences). Results are expressed as pg/mL of each cytokine ± SD of triplicate samples. ANOVA test was applied to the cytokine's concentrations according to the manufacturer recommendation (*P* ≤ 0.05). **(C)** Scheme of vaccination model and sample collection to analyze immune responses and protection: spleens and sera. **(D)** Vaccination of C57BL/6 mice with a single dose of DC vaccines. Seven days later, each group of vaccinated mice are divided in 3 sets and challenged i.v with 10^4^ CFU/mice of hypervirulent strains of HUMV-LM01, HUMV-MM01, or HUMV-SP01. Next, after 14 days mice are bled, sacrificed and spleens collected. Vaccination results expressed percentages of protection as the mean ± SD of triplicates. Percentages are calculated as the number of CFU/mL counted in spleen homogenates of NV mice (saline) divided by CFU/mL of each set of vaccinated mice. Results are expressed as the mean ± SD of triplicates. Student *t*-Test was applied for statistical analysis (*P* ≤ 0.05). CFU of non-vaccinated mice are the following: saline LM (HUMV-LM01) 2.75 × 10^5^ CFU/mL, DC-CONT LM (HUMV-LM01) 2.60 × 10^5^ CFU/mL, saline MM (HUMV-MM01) 1 × 10^5^ CFU/mL, DC-CONT MM (HUMV-MM01) 0.9 × 10^5^ CFU/mL, saline SP (HUMV-SP01) 2.5 × 10^5^ CFU/mL, DC-CONT SP (HUMV-SP01) 2.49 × 10^5^ CFU/mL.

There is another possible explanation for DC-LM-GAPDH_rec_ vaccines generating high DTH immune responses with induction of CD8^+^ and CD4^+^ T cells that is not related to the antigen immunogenicity. Some antigens can also induce DC activation, such as adjuvants or cell-walls of dead bacteria (16–19) and are interesting compounds for vaccine platforms. Here, we tested the possibility that LM-GAPDH_rec_, LM-LLO_rec_, or mRNA-LIPO complexes of LM-GAPDH or LM-LLO serve as non-specific DC activators. We evaluated two characteristics of activated DC, the cell surface expression of activation markers and the production of cytokines. We treated DC with different reagents, LM (HUMV-LM01), MM (HUMV-MM01), SP (HUMV-SP01), LM-LLO_rec_, LM-GAPDH_rec_, mRNA-LIPO complexes of LM-GAPDH or LM-LLO for 16 h, to examine activation. Two different adjuvants were also included in the assay, LPS and DIO-1 (14). Classical cell surface activation markers of DC are CD11c, MHC-II, CD40, or CD86, while CD11b is a macrophage-DC marker that, upon DC activation, reduces its surface expression and GR1 is a classical polymorphonuclear leukocyte (PMN) marker. LM (HUMV-LM01), MM (HUMV-MM01), and SP (HUMV-SP01) bacteria clearly induce DC activation, reflected by high percentages of CD11c, MHC-II, CD40, and CD86 positive cells (Figure 3A). mRNA-LIPO complexes of LM-GAPDH or LM-LLO did not induce DC activation, as the percentages of positive cells for MHC-II, CD40, or CD86 were similar to non-infected controls (NI). Recombinant LM-GAPDH_rec_ protein was the only antigen form that clearly increased the percentages of all DC activation markers, CD11c, MHC-II, CD40, and CD86. However, LM-GAPDH_rec_ effect was different than the activation pattern induced with LPS that increased only the percentages of the MHC-II activation marker (violet bars in Figure 3A) and was also different to the activation pattern induced by the DIO-1 adjuvant that increased the percentages of two activation markers, MHC-II and CD40. Neither LPS (dark blue bars), nor DIO-1 (garnet bars) caused significant effects in the percentages of CD86 positive cells. We conclude that LM-GAPDH_rec_ activation of DC affected the expression of all classical markers of DC activation (light blue bars), suggesting a broader activation pattern. Next, we explored other features of DC activation, after collection of DC supernatants and analysis of cytokines using a Th1-Th2 parametric flow cytometry assay (BD Biosciences). As shown in Figure 3B, DC stimulation with adjuvants as LPS released high levels of Th1 (MCP-1, TNF-α, or IFN-α and Th2 (IL-6 and IL-10) cytokines; while stimulation with adjuvants like DIO-1 produced Th1 (MCP-1, TNF- α, or IFN-α), but not Th2 cytokines. DC stimulation with mRNA-LIPO complexes of GAPDH or LLO produced no cytokine at all (undetectable levels) and LLO_rec_ only showed low levels of Th1 cytokines (1–5 pg/mL). DC stimulated with recombinant LM-GAPDH_rec_ released high levels of Th1 cytokines such as MCP-1, TNF-α, IFN-α, and IL-12, while no significant levels of Th2 cytokines such as IL-6 or IL-10 were observed. IL-12 production is associated with the ability to stimulate CD8^+^ T cells and might explain the effect of DC loaded with recombinant LM-GAPDH_rec_ to promote DTH responses (red bars in Figure 2C) after DC activation. We also confirmed that GAPDH_rec_ was able to activate monocyte derived DC (MoDC) from healthy donors, as they induced Th1 cytokines with high levels of IL-12 and very low levels of IL-6 and IL-10 (Supplementary Table 3). We conclude that LM-GAPDH_rec_ is a classical pro-inflammatory adjuvant that is able to activate DC in a stronger and broader manner.

### Validation of DC-GAPDH_rec_ as CRV Vaccines for *Listeria monocytogenes, Mycobacterium marinum*, and *Streptococcus pneumoniae* Infections

Specific DC activation with production of IL-12 have been linked to vaccine efficiency (47), therefore, we tested the vaccine efficiency of DC loaded with the highest immunogenic antigen forms, recombinant proteins LM-GAPDH_rec_ and LM-LLO_rec_ (see Figure 3C for vaccination scheme). Five mice per group were inoculated *i.v* with a single dose of DC vaccines (10^6^ cells/mice) pre-loaded with 5 μg/mL of LM-LLO_rec_ or LM-GAPDH_rec_ (DC-LM-LLO_rec_ or DC-LM-GAPDH_rec_) for 7 days and was then challenged *i.v* with either LM (HUMV-LM01), MM (HUMV-MM01), or SP (HUMV-SP01) for 14 days. Next, mice were sacrificed and their sera and spleens were collected. CFU were examined in spleens by plating in specific agar plates and results were expressed as the percentages of protection (see Material and Methods, section Vaccination Experiments With DC Vaccines Loaded With Listeria Recombinant Proteins or mRNA-LIPO for the detailed procedure). Only DC vaccines pre-loaded with LM-GAPDH_rec_ conferred good protection against a challenge with LM (HUMV-LM01), MM (HUMV-MM01), or SP (HUMV-SP01) (blue, red and green bars in Figure 3D), while DC-LM-LLO_rec_ protected only for LM (HUMV-LM01) infection. Empty DC showed no protection at all against any of the pathogens (bars labeled as DC-CONT in Figure 3D).

We also checked specific humoral and cellular immune parameters in vaccinated and non-vaccinated mice reported in experimental listeriosis vaccines (38, 48), such as the presence of antibodies recognizing the LM-GAPDH_1−22_ peptide in sera and the percentages of CD4^+^ or CD8^+^ cells specific for LM-GAPDH_1−22_ peptide-specific and IFN-γ producers, as well as verification of high frequencies of CD8^+^ T cells specific for GAPDH_1−22_ peptide using H2-Kb:Ig dimers (Table 2, see procedures in Materials and Methods, section Intracellular IFN-γ *Staining*). We detected high titers of antibodies recognizing LM-GAPDH_1−22_ epitope, and high percentages of GAPDH_1−22_ specific CD4^+^ and CD8^+^ and IFN-γ producers after vaccination with DC-LM-GAPDH_rec_ and being challenged with LM (HUMV-LM01), MM (HUMV-MM01), or SP (HUMV-SP01) infections. Moreover, these vaccinated mice presented very high frequencies of CD8^+^ T cells specific for the GAPDH_1−22_ peptide, while non-vaccinated mice challenged with LM, MM, or SP presented undetectable frequencies. We concluded that DC-LM-GAPDH_rec_ vaccines caused mainly antigen specific DC immune stimulation that confer cross-protection against LM, MM, and SP and induced GAPDH specific immune responses, both in T and B cells. However, we cannot discard non-specific broader DC immune stimulation.

**Table 2:** Specific immune responses elicited after vaccination of mice with DC-LM-GAPDHrec and challenge with LM (HUMV-LM01), MM (HUMV-MM01), or SP (HUMV-SP01).

**Mice vaccination^a^**	**^c^anti-GAPDH_**1−22**_ antibodies**	**^d^CD4^**+**^ %GAPDH_**1−22**_ and IFN-**γ****	**CD8^**+**^ % GAPDH_**1−22**_ and IFN-**γ****	**^e^% Gated dimer CD8/GAPDH_**1−22**_**
^b^HUMV-LM01 (NV)	0.85 ± 0.1	0.8 ± 0.2	0.7 ± 0.3	0.4 ± 0.1
HUMV-MM01 (NV)	0.75 ± 0.1	0.8 ± 0.2	0.4 ± 0.3	0.3 ± 0.1
HUMV-SP01 (NV)	0.67 ± 0.1	0.9 ± 0.2	0.3 ± 0.3	0.4 ± 0.1
DC-GAPDHrec/LM01	2.31 ± 0.2	2.1 ± 0.3	4.2 ± 0.2	4.5 ± 0.3
DC-GAPDHrec/MM01	2.10 ± 0.1	1.9 ± 0.2	3.9 ± 0.3	3.5 ± 0.2
DC-GAPDHrec/SP01	2.01 ± 0.1	2.1 ± 0.3	2.7 ± 0.2	3.5 ± 0.1
**CONTROL-NI**	**0.1** **±** **0.2**	**0.3** **±** **0.1**	**0.5** **±** **0.1**	**0.2** **±** **0.1**

a *Female C57BL/6 mice (n = 5) were i.v. vaccinated with DC loaded with GAPDH_rec_ and 7 days later they were challenged i.v with 5 × 10^3^ CFU bacteria from clinical isolates HUMV-LM01, HUMV-MM01, or HUMV-SP01. Fourteen days later, mice were bled, sacrificed and spleens collected*.

b *Mice non-vaccinated (NV) and challenged with the different pathogens are examined for anti-GAPDH_1−22_ in sera and peptide specific CD4 or CD8*.

c *Sera from mice as in a were examined for anti-GAPDH_1−22_ antibodies by a peptide ELISA. Results are presented as the mean ± SD of OD units in triplicate experiments. Student t-Test was applied for statistical analysis (P < 0.05)*.

d *Spleens of mice vaccinated or not were homogenized and cultured cells were used to measure intracellular IFN-γ after peptide stimulation in the presence of brefeldin A (procedure described in Material and Methods section Intracellular IFN-γ Staining). The percentages of CD4^+^ or CD8^+^ expressing IFN-γ were determined according with the manufacturer's recommendations. ANOVA test were applied for statistical analysis (P ≤ 0.05)*.

e *Some experiments spleen cells of vaccinated and non-vaccinated mice were incubated with recombinant dimeric H-2Kb:Ig fusion protein loaded with GAPDH_1−22_ peptide. The staining cocktail contained dimeric fusion protein loaded with GAPDH_1−22_ peptide, CD8, and IFN-γ antibodies. CD8^+^ were gated for anti- IFN-γ staining (%Gated dimer-CD8) to calculate the frequencies of CD8^+^-GAPDH_1−22_ restricted cells and IFN-γ producers. Results are the mean ± SD of triplicates. ANOVA test were applied for statistical analysis (P ≤ 0.05)*.

## Conclusion

*Listeria monocytogenes* GAPDH in two forms, either as a recombinant protein or as an mRNA-GNP complex, appears to be a safe bacterial antigen that induce significant T cell mediated immune responses when used in DC vaccine vectors. However, only the *Listeria* GAPDH recombinant protein activates DC in a specific and non-specific but broader form, different than adjuvant activation, as it induces all relevant activation markers and high production of Th1 cytokines, including IL-12. Therefore, not only is stimulation of T cell immune responses required for an antigen form to be considered a good candidate for vaccines, but specific DC activation also seems necessary to induce cross-protection against *Listeria, Mycobacterium*, and *Streptococcus* infections. DC vaccines loaded with recombinant LM*-*GAPDH can be considered not only as CRV vaccines with cross-protection abilities, but also as TIbV vaccines, since they present broad-spectrum protection for the common GAPDH virulence factor of *Listeria, Mycobacterium*, and *Streptococcus* and induces specific GAPDH immune responses. In fact, cross-protection abilities of these vaccines correlate with high levels of antibodies and high percentages of specific CD4^+^, CD8^+^ T cells, and IFN-γ producers, recognizing the N-terminal GAPDH_1−22_ peptide that has 98% homology in *Listeria, Mycobacterium*, and *Streptococcus*. The ability of mRNA-lipid carrier complexes to induce DC activation and strong T cell responses should be improved to include them in vaccine formulations for multivalent vaccines.

We speculate that experimental multivalent vaccines that can protect against *Listeria, Mycobacterium, Streptococcus*, bacterial genera responsible for severe meningitis, and long-lasting cutaneous infections in adults and the elderly, are promising tools for the new generation of human vaccines that are based on cross-reactive immunity, either as multivalent or as trained immunity-based vaccines.

## Patents

This study is protected by patent number WO200802108A1. https://patents.google.com/patent/WO2008020108A1/fi#patentCitations (accessed March 27, 2021), entitled to Fundación Marqués de Valdecilla and patent number WO2019243647. https://patentscope.wipo.int/search/es/detail.jsf?docId=WO2019243647 (accessed March 27, 2021), entitled to Fundación Instituto de Investigación Marqués de Valdecilla.

## Data Availability Statement

The original contributions presented in the study are included in the article/Supplementary Material, further inquiries can be directed to the corresponding author/s.

## Ethics Statement

The studies involving human participants were reviewed and approved by Committee of Clinical Ethics of Cantabria. The patients/participants provided their written informed consent to participate in this study. The animal study was reviewed and approved by Universidad de Cantabria Animal Ethical Committee.

## Author Contributions

CA-D designed the experiments and directed the coordination of the experiments. DS-C and HT-N performed all the experiments and contributed equally to this study. RC-G helped in the performance of the vaccination experiments. RT performed the bioinformatic analyses. JC-M and IP-D provided all the clinical isolates, collected sera from patients, collected the Informed Consents of the patients, and have the custody of them. SY-D provided clinical isolates and clinical histories of patients infected with *M. marinum*, collected the Informed Consents of the patients and have their custody and helped with the mice models of infection. All authors contributed to the article and approved the submitted version.

## Conflict of Interest

MF was employed by company DIOMUNE S.L. The remaining authors declare that the research was conducted in the absence of any commercial or financial relationships that could be construed as a potential conflict of interest.
