# Supplementary material for: A Comparison Between Recombinant Listeria GAPDH Proteins and GAPDH Encoding mRNA Conjugated to Lipids as Cross-Reactive Vaccines for Listeria, Mycobacterium, and Streptococcus
Source: Front Immunol. 2021 Apr 19;12:632304. doi: 10.3389/fimmu.2021.632304 (PMC8092121; doi:10.3389/fimmu.2021.632304)
Supplement: Supplementary file 1 [file Data_Sheet_1.zip › Supplementary Material Data Sheet 1.pdf]

Table S1. Protein colour aligments of Figure 1B.

This protein-only option colours the residues according to their physicochemical properties:

Residue	Colour	Property
AVFPMILW	RED	Small (small+ hydrophobic (incl.aromatic -Y))
DE	BLUE	Acidic
RK	MAGENTA	Basic - H
STYHCNGQ	GREEN	Hydroxyl + sulfhydryl + amine + G
Others	Grey	Unusual amino/imino acids etc

Table S2. Virulence in mice of the different clinical isolates collected in year 2016.

**Isolate code[a] Year 2016**	**Virulence in mice (CFU/mL)[b]**
*HUMV-LM01	$2.9 \times 10^5 \pm 10$
HUMV-LM02	$2.7 \times 10^5 \pm 11$
HUMV-LM03	$2.5 \times 10^5 \pm 12$
HUMV-LM04	$2.2 \times 10^5 \pm 12$
HUMV-LM06	$2.2 \times 10^5 \pm 10$
HUMV-LM07	$2.4 \times 10^5 \pm 11$
LM^{WT} (10403S basal control)	**$2.5 \times 10^2 \pm 12$**
LM-ΔLLO (non-virulent control)	**$4.2 \times 10^0 \pm 10$**
HUMV-MTB01	$3.9 \times 10^4 \pm 10$
HUMV-MA01	$3.5 \times 10^4 \pm 12$
*HUMV-MM01	$3.8 \times 10^4 \pm 11$
HUMV-MC01	$3.0 \times 10^4 \pm 10$
HUMV-MC02	$3.0 \times 10^4 \pm 11$
HUMV-MC03	$3.2 \times 10^4 \pm 12$
HUMV-MC04	$3.0 \times 10^4 \pm 11$
HUMV-MC05	$3.1 \times 10^4 \pm 11$
M. smegmatis (non-virulent control)	**$1.5 \times 10^2 \pm 8$**
*HUMV-SP01	$3.8 \times 10^5 \pm 13$
HUMV-SP02	$3.5 \times 10^5 \pm 16$
HUMV-SP03	$3.3 \times 10^5 \pm 15$
HUMV-SA01	$4.8 \times 10^5 \pm 13$
HUMV-SA02	$4.9 \times 10^5 \pm 12$
HUMV-SA03	$4.8 \times 10^5 \pm 11$
HUMV-SPY01	$4.1 \times 10^5 \pm 17$
HUMV-SPY02	$4.0 \times 10^5 \pm 12$
HUMV-SPY03	$4.2 \times 10^5 \pm 12$
S. pneumoniae 49619-19F (vaccine control)	**$1.2 \times 10^3 \pm 10$**
**CONTROL-NI**	**0.06 ± 0.1**

[a]Clinical isolates in the year 2016 from patients older than 50 years of age and infected with the following strains of *L. monocytogenes* (*HUMV-LM01-HUMV-07, 7 isolates), different mycobacteria (M. *tuberculosis*, HUMV-MTB01; *M. avium*, HUMV-MA01; *M. marinum*, *HUMV-MM01 and *M. chelonae*, HUMV-MC01-HUMV-05) or different streptococci (*S. pneumoniae*, *HUMV-SP01-HUMV-SP03; *S. agalactiae*, HUMV-SA01-HUMV-SA03 and *S. pyogenes*, HUMV-SPY01-HUMV-SPY03). Non-pathogenic LM-ΔLLO, *M. smegmatis* and *S. pneumoniae* 49619-19F strains were also included in the assay as non-virulent controls. [b]Female C57BL/6 mice (n = 5) were *i.v.* inoculated with 10^4 CFU/mice of the clinical isolates detailed in *a*. Fourteen days later, mice were bled, sacrificed and spleens collected. Spleens were homogenized and triplicate of serial 1/10 dilutions plated in agar plates. CFU were counted and results expressed as CFU/mL. Student t test was applied for statistical analysis ($P \leq 0.5$). Asterisks and yellow highlighted data correspond to the selected clinical isolates for our study.

Table S3.- Adjuvant abilities of recombinant *Listeria* proteins using MoDC of healthy donors.

[a]Condition	[b]TNF-α	IFN-α	IL-12	IL-6	IL-10
**NT**	10 ± 0.3	4 ± 0.3	1 ± 0.1	3 ± 0.2	3 ± 0.1
**LLO_{rec}**	100 ± 0.2	10 ± 0.2	1 ± 0.1	3 ± 0.2	5 ± 0.2
**$GAPDH_{rec}$**	550 ± 0.6	450 ± 0.3	48 ± 0.4	3 ± 0.1	4 ± 0.1
**LPS**	623 ± 0.6	330 ± 0.4	1 ± 0.1	100 ± 0.5	100 ± 0.5
**DIO-1**	600 ± 0.7	450 ± 0.6	5 ± 0.1	35 ± 0.4	10 ± 0.3

[a]MoDC were incubated for 16 hours in the presence of 5 µg/mL of the different reagents: recombinant proteins LLO, GAPDH or adjuvants LPS or DIO-1. Culture supernatants were collected and stored at -80ºC until use. [b]Cytokines are measured by flow cytometry using the CBA kit of Becton Dickinson (BS Biosciences, San Jose, CA, USA). Cytokine concentrations are expressed as pg/mL. Data are analyzed with FlowJo software. ANOVA test was applied to the samples according to manufacturer's instructions.

FIGURE LEGEND

Figure S1.- Quality controls of mRNA-lipid carrier complexes and recombinant antigens and their toxicities examined in macrophages and DC. ***A,*** Concentration of the mRNA preparations and quality analysis performed by absorbance measurements. Results were performed in triplicate and they show the ranges of quality assays performed. ***B,*** Toxicities of mRNA-lipid carrier complexes or recombinant proteins into macrophages (BM-DM) or DC (BM-DC) were explored analyzing the abilities of cell lysates to hemolyze sheep-red-blood cells in a procedure previously described [2]. Controls include cells infected with the different pathogens, LM (HUMV-LM01), MM (HUMV-MM01) or SP (HUMV-SP01). Results are the mean ± SD of triplicates. Student t test was applied for statistical analysis. ($P \leq 0.5$). ***C,*** Toxicities of mRNA-lipid carrier complexes or recombinant proteins into DC were also explored by Trypan blue experiments. Results are the mean ± SD of triplicates. Student t test was applied for statistical analysis. ($P \leq 0.5$)
