# Supplementary material for: A Comparison Between Recombinant Listeria GAPDH Proteins and GAPDH Encoding mRNA Conjugated to Lipids as Cross-Reactive Vaccines for Listeria, Mycobacterium, and Streptococcus
Source: Front Immunol. 2021 Apr 19;12:632304. doi: 10.3389/fimmu.2021.632304 (PMC8092121; doi:10.3389/fimmu.2021.632304)
Supplement: Supplementary file 1 [file Data_Sheet_1.zip › Supplementary Material Figure 1.pdf]

Figure S1

A

Antigen	mRNA concentration	Protein/RNA quality (260/280 ratio ≥ 1,6)	RNA/DNA quality (260/230 ratio ≥ 1)
LLO-LM	5,2 ng/µL	2,05-2,13	1,45-1,6
GAPDH-LM	4,8 ng/µL	1,86-2,03	2,08-2,17
Ag85A-MM	5,6 ng/µL	2,07-2,10	1,95-2,08
PLY-SP	4,1 ng/µL	1,87-2,05	1,29-1,58

B

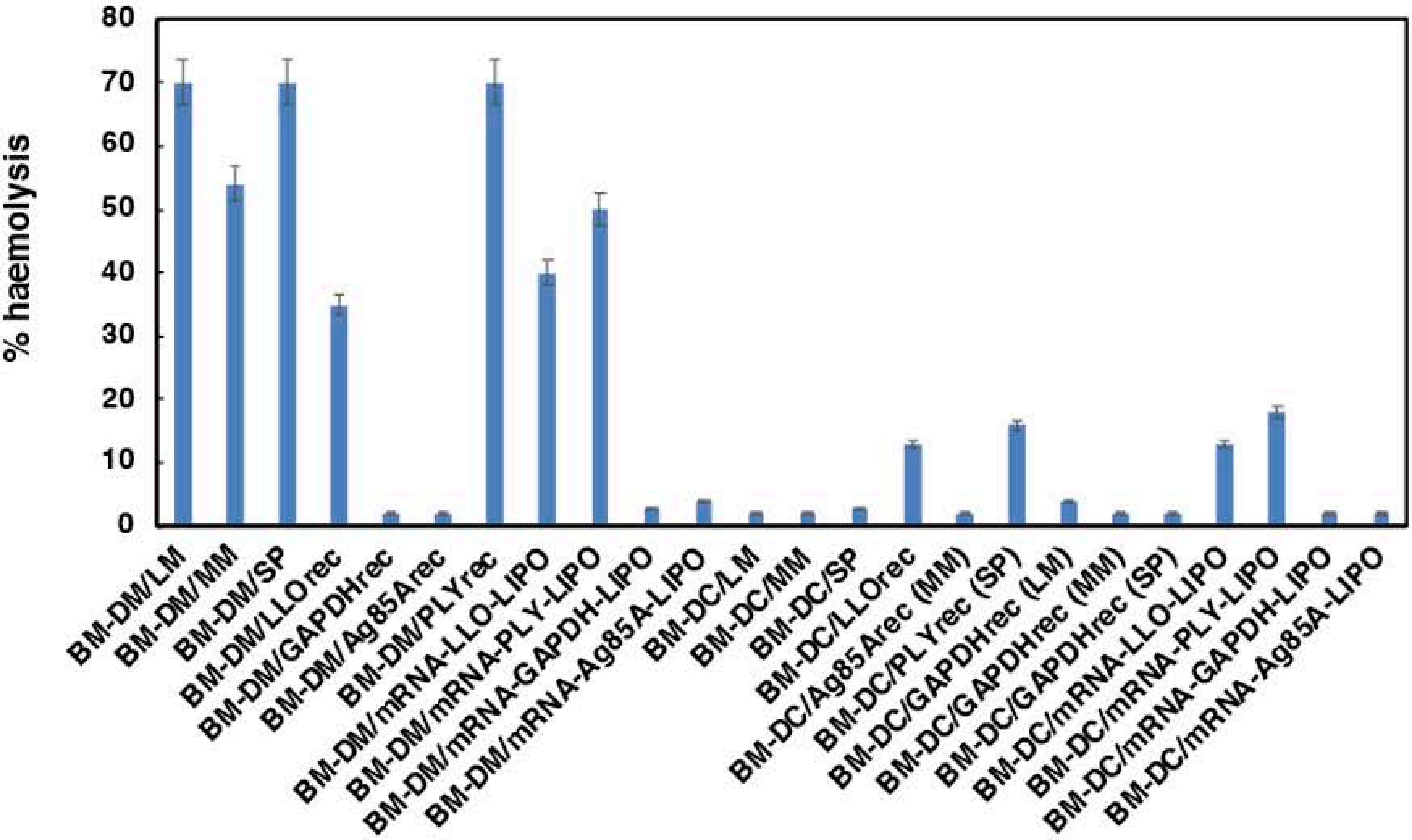

C

Samples tested	*Cell viability (% Trypan blue⁺ cells)
DC + saline	2.0% ± 0.3
DC + LLOrec	1.5% ± 0.6
DC + [a]mRNA-LLO-LIPO	1.4% ± 0.4
DC + mRNA-GAPDH-LIPO	3.0% ± 0.5
DC + GAPDHrec	2.9% ± 0.7
DC + mRNA-PLY-LIPO	2.9% ± 0.7
DC + PLYrec	1.5% ± 0.6
DC + mRNA-Ag85A-LIPO	2.9% ± 0.7
DC + Ag85Arec	2.45 ± 0.5
DC + LIPO	2.68 ± 0.4

*cell viability is stained with Trypan blue that accumulates in nuclei and cytoplasm in non-viable cells.

[a]LIPO means lipofectamine
